# The Effects of Different Re-Warm-Up Strategies on Power, Changing of Direction and Ball Shooting Velocity in Well-Trained Soccer Players

**DOI:** 10.3390/sports11090169

**Published:** 2023-09-05

**Authors:** Demetris Matsentides, Marios Christou, Nikolaos Zaras

**Affiliations:** 1 Human Performance Laboratory, Department of Life Sciences, School of Life and Health Sciences, University of Nicosia, Nicosia 1700, Cyprus; d.matsentides8@gmail.com (D.M.); christoumar@gmail.com (M.C.); 2 Sports Center, University of Cyprus, Nicosia 1700, Cyprus; 3 Cyprus Sports Organization, Nicosia 2400, Cyprus

**Keywords:** change of direction, vertical jump, ball shooting velocity, warm-up

## Abstract

The purpose of the study was to investigate whether a re-warm-up training session either with tuck jumps and linear sprints or with changing of directions may enhance power, agility or ball shooting velocity in well-trained soccer players. Ten soccer players (age: 18.2 ± 1.7 years; body mass: 64.4 ± 8.0 kg; body height: 1.71 ± 0.04 m) participated in the study. Players performed three different re-warm-up interventions including no re-warm-up (C), change of direction (COD) and jump-sprint condition (JS). Before each re-warm-up intervention, players performed the same warm-up condition followed by 8 min of passive rest. Following the re-warm-up interventions, countermovement jump (CMJ), T-Test agility time-trial and ball shooting velocity were measured. Performance in CMJ height, power and power per body mass remained unchanged following all three conditions (*p* > 0.05). However, the agility time-trial was significantly reduced following COD re-warm-up compared to C (−1.7 ± 1.6%, *p* = 0.03). Ball shooting velocity was increased following COD compared to C (4.7 ± 3.8%, *p* = 0.014), while a statistical trend was found between JS and C interventions (4.8 ± 5.4%, *p* = 0.060). These results suggest that a re-warm-up intervention including changing of directions may significantly enhance T-Test agility time-trial and ball shooting velocity in well-trained soccer players.

## 1. Introduction

Soccer is considered one of the most popular sports in the world. Performance in soccer depends in several factors including shooting and passing the ball, dribbling the ball, sprinting, jumping and rapid changing of directions [1]. Thus, it is crucial for coaches and strength and conditioning professionals to design effective warm-up sessions before training or prior to a soccer game in order to maximize performance in the playing field. Warm-up may lead to significant increases in muscle and core temperature [2,3], as well as in higher neuromuscular activation mainly by the presence of the post-activation performance enhancement (PAPE) phenomenon, which may lead to a short-term increase in performance [4,5,6]. Results from two scientific reviews showed that the ideal duration of warm-up in team sports ranged between 10 to 15 min, which appears to induce greater enhancements in competitive performance compared to longer warm-up programs [7,8]. However, a usual problem for team sports and especially for soccer is the rest intervals between the initial warm-up and the beginning of the game and/or the half-time. During this period, players receive the last tactical instructions from the coaching team, replenish fuel stores, partially recover from the high intensity warm-up or the first half, and potentially receive minor medical treatment. Previous study has shown that half-time passive rest may lead to a significant decrease in muscle and core temperature, leading to a significant reduction in sprinting performance [2], while for every 1 °C reduction in muscle temperature, there is an additional 3% reduction in lower-body power output [9]. Consequently, coaches and strength and conditioning professionals apply a re-warm-up training session, which can inhibit the negative effects of passive rest before entering the game or during half-time [10].

In contrast to the basic warm-up, re-warm-up is a short duration training session where players usually perform high or low intensity exercises in order to regain the positive effects of warm-up [7]. Re-warm-up is applied minutes prior to the initiation of the soccer game or during half-time. Although several studies have focused on the effects of re-warm-up during half time [2,11,12,13], a handful of studies have investigated the effect of re-warm-up during the beginning of the soccer game. A study in 22 Portuguese elite under-19 football players examined the effects of four different re-warm-up activities before the initiation of the first half, 6 min following the basic warm-up. The results of the study showed that repeated changes of direction and plyometrics re-warm-up interventions had beneficial effects to vertical jumps (3.5% and 3.8%, respectively) and linear sprints (−2.8% and −3.4%, respectively), while eccentric re-warm-up had a harmful effect, especially in vertical jumps [14]. In a recent study, 23 high-level soccer players (under 17 years of age) followed a half-time short re-warm-up strategy either with passive rest or with 3 min of light running, skipping, jumping–sprinting and changing of directions exercises. The study showed that the 3 min re-warm-up strategy maintained performance in jumping, linear sprinting and changing of directions compared to post-warm-up [15]. Whether a short re-warm-up strategy may enhance changing of direction prior to the initiation of a soccer game needs further investigation.

One crucial parameter for high performance in soccer is shooting and passing the ball. The ability to shoot the ball faster combined with accuracy may increase the likelihood of achieving a goal [16]. A previous study has shown that ball velocity was correlated with both anthropometric and physiological factors, leading to the conclusion that increasing sprint velocity, vertical jump, agility and lower body strength may improves players’ kicking ability [17]. Indeed, long-term training studies have shown that ball shooting velocity may be increased following resistance training, plyometrics and sprint training [16,18,19]. However, the data are scarce regarding the effects of warm-up on ball shooting velocity and, especially, whether a re-warm-up short-term training session may enhance shooting velocity.

Therefore, the purpose of the current study was to investigate the effects of short-term re-warm-up interventions on CMJ, changing of direction and ball shooting velocity in soccer players. The hypothesis was that re-warm-up interventions will enhance all fitness attributes compared to no re-warm-up.

## 2. Material and Methods

### 2.1. Participants

Fourteen male soccer players initially volunteered to participate in the study. However, four players withdrew from the experimental procedures for purposes unrelated to the study. Consequently, 10 soccer players [age: 18.2 ± 1.7 years (age ranged: 16–20 years); body mass: 64.4 ± 8.0 kg; body height: 1.71 ± 0.04 m, with 11.3 ± 2.8 years of training experience participated in the study. Players were recruited from three different soccer clubs competing in the U-19 and Men’s second division Cypriot League. From the total of 10 players, four were forwards and six were defenders, while all of them were among the starter players for their team during competitions. For being eligible to participate in the study, players should meet the following criteria: absence of drug abuse or any illegal nutritional supplements, regular participation in soccer training and official games during the two previous years, and free from any cardiovascular diseases and musculoskeletal injuries. Athletes were fully informed about the experimental procedures and signed an informed consent. For players under the age of 18, a parental consent form was obtained. All procedures were in accordance with the 1975 Declaration of Helsinki as revised in 2013 and were approved by the National Bioethics Committee (EEBK/ΕΠ/2020/55).

### 2.2. Experimental Design

**Figure 1:**
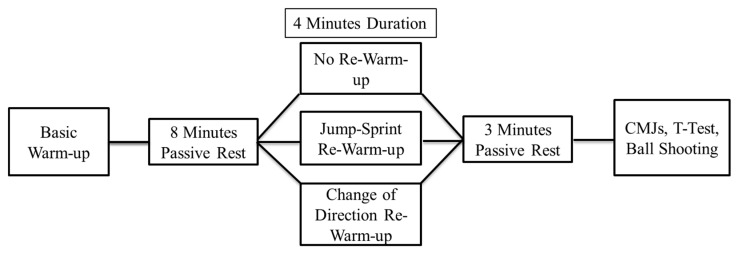
Experimental design of the study.

A crossover-controlled study was conducted during the competitive season. Players followed three different re-warm-up interventions including no re-warm-up (C), changing of direction (COD) and jump–sprint condition (JS). In order to achieve realistic game conditions, we hypothesized that players would need approximately 15 min after the warm-up to return to the locker rooms, change clothes, take last coaching instructions and return to the field for team presentation before the beginning of the soccer game. Each re-warm-up intervention had an approximately 4 min duration and was performed 8 min [2,13] after the same standardized warm-up. All re-warm-up conditions were randomly performed during a 10 day experimental period with at least 72 h between interventions (Figure 1). Players visited the training facilities on four different occasions. During the first day, anthropometric characteristics and a familiarization training session including the re-warm-up conditions as well as countermovement jumps (CMJ), T-Test agility time trial and ball shooting velocity measurements were performed in that exact order. Then, during the next three experimental days, players were randomly assigned into three different groups and followed the re-warm-up interventions. Immediately after the re-warm-up interventions, players performed the CMJs, 1 min after players performed the T-Test agility time trial and 2 min later players performed the ball shooting velocity measurement. At the end of the experimental procedures, all players completed the three different re-warm-up interventions. Changes in CMJ, T-Test agility time trial and ball shooting were compared between C, COD and JS.

### 2.3. Training

Re-warm-up interventions were performed during the competition period. At the time that the study was conducted, players performed approximately 5 training sessions per week, including soccer drill training, cardiovascular endurance exercise, resistance training and plyometrics. In addition, players participated at least in one official soccer game every weekend. In order to minimize circadian influences, all re-warm-up interventions and tests were performed at the same time of the day. Players followed the same warm-up before applying the re-warm-up interventions. Warm-up consisted of running, dynamic stretching, agility-stair exercises, sprinting exercises and ball shooting. After the basic warm-up, a 15 min experimental procedure followed (Figure 1). An 8 min passive rest was placed between the basic warm-up and re-warm-up interventions. The duration of COD and JS re-warm-up interventions had approximately 4 min duration. Consequently, we aimed to provide additional 3 min [20] of rest so that players could benefit from the PAPE phenomenon and reduce fatigue, since re-warm-up conditions were performed with maximum intensity [20,21]. More specifically, for the C condition, players walk for 4 min at the side line of the soccer field at low intensity at approximately 70–80 bpm. For the JS condition, players performed 4 maximum tuck jumps and then a maximum 20 m linear sprint. Players performed 4 repetitions with 40 s rest between repetitions. For the COD condition, players performed 4 repetitions of maximum velocity sprints with 180° change of direction every 5 m covering a 20 m total distance with 40 s rest between repetitions. Players were instructed to sprint and accelerate and decelerate as fast as possible. During both re-warm-up interventions, a certified strength and conditioning researcher was present to vocally encourage players to perform their maximum efforts. The vocal encouragement was exactly the same for all players.

### 2.4. Anthropometric Characteristics and Familiarization Session

Body mass and body height were measured during the first day of the experimental procedures. Soccer players were instructed to wear their training uniforms except for their shoes and socks. Body mass was calculated to the nearest 0.1 kg with an electronic scale, and body height was measured to the nearest 0.1 cm with a stadiometer (Tanita, VB 3000, Manchester, UK). Then, players performed the same warm-up (see above) and performed a familiarization session with all the performance measurements. More specifically, players performed 5 to 6 CMJ’s, 3 to 4 T-Test time trials with progressively increasing velocity and numerous ball shooting attempts from the penalty spot. At the end of the familiarization session, soccer players followed a 5 min cool-down with light running and static stretching.

### 2.5. Countermovement Jump

The first performance test following the re-warm-up interventions was the CMJ (OptoJump Next, Microgate, Bolzano, Italy). Players performed 3 maximum CMJs with arms akimbo while 45 s rest was given between attempts. During CMJs, players had the instruction to jump as high as possible. Following each jumping attempt, researchers informed players about the jump height (cm) in order to motivate them for a greater effort. The mean CMJ height from all 3 attempts was used for the statistical analysis [22]. Power and power relative to body mass were also calculated [23]. The intra class correlation coefficient (ICC) for CMJ height, power, and power relative to body mass were 0.989 (95% confident intervals (CI): Lower = 0.957, Upper = 0.997), 0.980 (95% CI: Lower = 0.985, Upper = 0.990), and 0.981 (95% CI: Lower = 0.978, Upper = 0.991), respectively.

### 2.6. T-Test Agility Time-Trial

After the CMJs, players performed the T-test agility time-trial. Briefly, one photocell gate (Witty, Microgate, Bolzano, Italy), was set at starting line approximately 1 m above the ground and 3 m apart, while players initiated their effort 0.5 m behind the starting line. Players had the instruction to run as fast as possible and maintain a facing position. For players who failed to maintain a facing forward position, crossed their feet or failed to touch a cone during their attempt, then the current attempt immediately stopped and players were given an extra attempt [24]. Two attempts were allowed with 2 min of rest, and the mean score of both attempts was used for statistical analysis. The ICC for the T-Test was 0.940 (95% CI: Lower = 0.820, Upper = 0.960).

### 2.7. Ball Shooting Velocity

After the T-Test agility time-trial, players performed the ball shooting velocity test with a 5 step approach. Shooting velocity was measured with a radar gun (Bushnell Velocity Sports Radar Gun, Kansas, USA) measuring to the nearest 1.0 km·h^−1^ [17], while all shots were performed with a standard game ball (mass 450 g and circumference 70 cm). Ball shooting was performed from the penalty spot (11 m from target). Players used the 5 step approach, and they shot the ball with the preferred leg [25] as fast as possible. The radar gun was positioned behind the goal, while all players were instructed to aim the ball between a target marked in front of the radar gun. Three to four warm-up shots were given, and then 5 maximum shots with 45 s of rest were allowed. The mean score of all ball shooting attempts was used for the statistical analysis. The ICC for the ball shooting velocity test was 0.970 (95% CI: Lower = 0.900, Upper = 0.990).

### 2.8. Statistical Analysis

All variables are presented as mean ± SD. Following the Shapiro–Wilk test, all data were normally distributed. A 3-way analysis of variance for repeated measures was used to examine differences between control, sprint and agility re-warm-up strategies. Because of the small sample size, Hedge’s g effect size was calculated with the following criteria used to infer the magnitude of the difference: <0.2 (trivial), 0.2–0.5 (small), 0.5–0.8 (moderate), and >0.8 (large). The reliability of all measurements was determined with a 2-way random effect intra-class correlation coefficient and confidence intervals. Significance was set at *p* ≤ 0.05.

## 3. Results

All players completed the experimental procedures without injuries. Table 1 presents the results from the CMJ variables, T-Test agility time-trial and ball shooting velocity. No significant differences were found for CMJ height (F_(2, 8)_ = 2, 706), power (F_(2, 8)_ = 2, 693) and power relative to body mass (F_(2, 8)_ = 2, 591) between the three different re-warm-up conditions.

A significant main effect was found for T-Test agility time-trial (F_(2, 8)_ = 4, 743, *p* = 0.044). More specifically, T-Test time trial was significantly reduced following COD re-warm-up compared to C (mean percentage difference: −1.7 ± 1.6%, *p* = 0.030, g = 0.184, 95% CI: Lower = 0.017, Upper = 0.321). However, no significant difference was found between JS and C (mean percentage difference: −1.2 ± 1.8%, *p* = 0.233, g = 0.130, 95% CI: Lower = −0.287, Upper = 0.055) or between COD and JS (mean percentage difference: −0.5 ± 1.6%, *p* = 0.989, g = 0.055, 95% CI: Lower = −0.102, Upper = 0.208).

A significant main effect was also found for ball shooting velocity (F_(2, 8)_ = 7, 036, *p* = 0.017). Specifically, ball shooting velocity was significantly increased following COD re-warm-up compared to C (mean percentage difference: 4.7 ± 3.8%, *p* = 0.014, g = 0.459, 95% CI: Lower = −7.740, Upper = −0.940). Moreover, a significant trend with moderate effect size was found between JS and C (mean percentage difference: 4.8 ± 5.4%, *p* = 0.060, g = 0.469, 95% CI: Lower = −8.980, Upper = 0.250). No significant difference was found between COD and JS (mean percentage difference: −0.1 ± 2.3%, *p* = 0.990, g = 0.015, 95% CI: Lower = −1.992, Upper = 2.286).

## 4. Discussion

The purpose of the study was to investigate the effects of re-warm-up either with tuck jumps and linear sprints or changing of directions on CMJ, T-Test agility time-trial and ball shooting velocity in well-trained soccer players. The main finding of the study was that the COD re-warm-up condition induced significant enhancement in T-Test agility time-trial and ball shooting velocity compared to no re-warm-up. Although no significant difference was observed between JS and C for T-Test agility time-trial, a statistical trend and a moderate effect size showed that ball shooting velocity may be enhanced following JS re-warm-up intervention compared to C. However, no significant difference was found for CMJ variables between all re-warm-up conditions. These results suggest that a 4 min re-warm-up training strategy including 20 m of repeated changing of directions may enhance T-Test agility time-trial and ball shooting velocity prior to a soccer game. In addition, a combination of tuck jumps and linear sprints may induce positive enhancements in ball shooting velocity, while it will not adversely affect changing of direction performance. Consequently, coaches and strength and conditioning professionals may apply a 4 min re-warm-up training session 8 min after completing the basic warm-up.

T-Test agility time-trial was significantly enhanced following COD re-warm-up compared to C, while no significant difference was found between COD and JS, as well as between JS and C. Changing of direction is considered to be a vital component for all team sports [26], while is essential for soccer, since players performed more than 700 direction changes during a game of varying intensity, and roughly 50 of the direction changes are performed at maximal intensity [24,27]. This finding may partially depend on the principle of specificity, since players performed a re-warm-up based on changing of directions, consequently it would be logical to assume that the T-Test agility time-trial would be improved [22,28]. Similar to the current results, re-warm-up strategies including repeated changing of directions and plyometric exercises before entering the soccer game had positive effects on linear sprint performance in young elite soccer players [14]. Another study showed that dynamic stretching and low-intensity isometric strength exercise may also enhance changing of direction compared to no re-warm-up [29]. However, in the latter study, the modified T-Test was applied (5 m × 5 m) while there was only 1 min of rest between the general warm-up and the re-warm-up session, and 2 min from re-warm-up to measurements. In the current study, a longer transition period (8 min) was allowed between the general warm-up and re-warm-up session, which might have reduced the muscle and core temperature. Moreover, 3 min of active rest was given to players following both re-warm-up interventions in order to rest and have greater benefits from the PAPE phenomenon. In addition, several studies that used re-warm-up during half-time have showed positive effects on linear sprint performance [2,11,12,13], although data are scarce regarding the effects on changing of direction. Consequently, coaches may apply a COD re-warm-up strategy in order to enhance changing of direction prior to the beginning of a soccer game.

An interesting finding of the study was that COD re-warm-up significantly increases ball shooting velocity compared to C, while there was a statistical trend (*p* = 0.060) for JS to increase ball shooting velocity compared to C. Shooting and passing the ball are essential factors for soccer performance, while any enhancements in ball velocity may increase the possibilities of goal achievement [16]. Although several studies have showed that ball shooting velocity is increased following long-term training programs [16,18,19] the effects of a short-term re-warm-up training session in ball velocity remained unexplored. Ball shooting depends on whole body power and fast force production; consequently, the positive acute effects of PAPE may lead to a significant neuromuscular activation following both re-warm-up interventions. Although accuracy was not evaluated, coaches should consider that higher ball shooting velocity may also show the level of players’ preparedness prior to the game. Still, more research is needed in order to reach certain conclusions.

Performance in CMJ remained unaltered following both re-warm-up interventions compared to C. This finding is in contrast to previous studies in the scientific literature, since CMJ was enhanced prior to the game [14] and following half-time [11,12]. In addition, a recent study showed that squat jump height maintained in similar levels to post-warm-up after a 3 min re-warm-up strategy in young soccer players [15]. These ambiguous results may be explained from different re-warm-up strategies and the fitness status of the players. In the current study, we can only speculate that either the basic warm-up was too exhausting for players, or the 3 min rest following the re-warm-up interventions may have not been enough time so that the positive benefits of PAPE would appear [30]. However, both re-warm-up interventions maintain performance in CMJ, while the non-significant differences of 2.3% between JS and C, as well as 2.1% between COD and C, should not be unnoticed by coaches. It should also be noted that the influence of each maximal performance test in the subsequent test might potentially have an impact on players’ overall performance. Consequently, performance in the T-Test agility time-trial and ball shooting velocity might be positively influenced by CMJs; the latter was performed first in the current study. Although this is a real world study, more research is needed that focuses on the positive or negative effect of the order of performance tests on the overall performance of soccer players.

The current study has some limitations. The small sample size and the different playing position of players might influence the standard deviations of variables. Moreover, the generalization of the results in elite soccer players may be limited. In addition, measurements were performed during the competitive period, where the fatigue from the repeated games was not evaluated. Muscle and core temperature were not measured, which might have provided better insights into the nature of the results. In addition, it is unknown how long these benefits from COD re-warm-up will last during the soccer game. However, one novelty of the current study was that measurements included the mean values of all attempts of soccer players, which might be a better indicator of the neuromuscular condition of players [22].

## 5. Conclusions

A short duration re-warm-up (4 min) consisting of repeated changing of directions maintain CMJ and enhance T-Test agility time-trial as well as ball shooting velocity compared to the absence of re-warm-up. In addition, a repeated tuck jump and linear sprint re-warm-up preserve CMJ, T-Test agility time-trial and possibly increase ball shooting velocity compared to no re-warm-up. Coaches and strength and conditioning practitioners may apply a changing of direction re-warm-up in well-trained soccer players 3 min before the initiation of the official game in order to enhance the neuromuscular performance of players.

## Figures and Tables

**Table 1:** Results for countermovement jump variables, T-Test time-trial and ball shooting velocity following the control, change of direction and jump and sprint re-warm-up interventions.

Variable	C	COD	JS	*p*-Value
CMJ height (cm)	34.0 ± 3.3	34.8 ± 4.1	34.9 ± 4.1	0.127
CMJ power (W)	2909.4 ± 428.0	2949.2 ± 464.2	2953.2 ± 460.3	0.128
CMJ power relative to body mass (W·kg^−1^)	45.1 ± 2.6	45.7 ± 3.2	45.8 ± 3.2	0.136
T-Test Time Trial (s)	10.45 ± 0.68	10.31 ± 0.79 *	10.37 ± 0.77	0.044
Ball Shooting Velocity (m·s^−1^)	94.4 ± 7.4	98.7 ± 7.9 *	98.9 ± 8.0	0.017

CMJ = countermovement jump, C = no re-warm-up, COD = change of direction condition, JS = jump sprint condition. * = Significant difference between COD and C.

## Data Availability

The data presented in this study are available on request from the corresponding author.
